# Contrast Sensitivity Impairment Predicts Decline in Balance Over 30 Months in Cognitively Normal Older Adults

**DOI:** 10.1093/geroni/igaf122.079

**Published:** 2025-12-31

**Authors:** Atalie Thompson, Ashley Wells, Michael Miller, Haiying Chen, Paul Laurienti, Stephen Kritchevsky

**Affiliations:** Wake Forest University School of Medicine, Winston-Salem, North Carolina, United States; Wake Forest University School of Medicine, Winston-Salem, North Carolina, United States; Wake Forest University School of Medicine, Winston-Salem, North Carolina, United States; Wake Forest University School of Medicine, Winston-Salem, North Carolina, United States; Wake Forest University School of Medicine, Winston-Salem, North Carolina, United States; Wake Forest University School of Medicine, Winston-Salem, North Carolina, United States

## Abstract

Contrast sensitivity impairment (CSI) increases in prevalence as older adults age, and may impact different aspects of physical performance over time. We conducted a single center prospective cohort study of 192 cognitively unimpaired older adults with good visual acuity and self-reported visual function. Linear mixed models examined the difference in the association of moderate CSI (log CS < 1.55) at baseline with standing balance time(seconds), 4m gait speed(m/s), narrow walking speed(m/s) and chair pace(stands/s) over 30 months. Multivariable models adjusted for the effect of age, race, and sex on the slopes of each type of physical performance. At baseline, the mean participant age was 76.5±4.7 years, with 56.5% (N = 108) female and 9.4% (N = 18) black. CSI predicted a significantly greater decline in balance time (Beta:-4.33, 95% CI (-7.41, -1.24), p = 0.006) relative to those with non-impaired CS (Beta:-1.05, 95% CI(-2.05, -0.04), p = 0.042), with a difference in slopes of –3.28 (95% CI (-6.53, 0.03), p = 0.048). The difference in slopes remained significant with adjustment for age, sex, and race effects (Beta:-3.51, 95% CI(-6.83, -0.20), p = 0.038). Those with CSI also experienced significant declines in 4m gait speed and narrow walking speed (p < 0.05), but the difference in slopes was not significant compared to those without CS impairment (p > 0.05), and there was no significant relationship of CSI with chair pace over time. In cognitively intact older adults with good visual acuity, CSI was associated with a significantly faster decline in balance time over 30 months of follow-up. CS testing may identify older adults at risk for developing postural instability.

